# Evaluating Chatbot responses to patient questions in the field of glaucoma

**DOI:** 10.3389/fmed.2024.1359073

**Published:** 2024-07-09

**Authors:** Darren Ngiap Hao Tan, Yih-Chung Tham, Victor Koh, Seng Chee Loon, Maria Cecilia Aquino, Katherine Lun, Ching-Yu Cheng, Kee Yuan Ngiam, Marcus Tan

**Affiliations:** ^1^Department of Ophthalmology, National University Hospital, Singapore, Singapore; ^2^Centre of Innovation and Precision Eye Health, Department of Ophthalmology, Yong Loo Lin School of Medicine, National University of Singapore and National University Health System, Singapore, Singapore; ^3^Singapore Eye Research Institute, Singapore National Eye Centre, Singapore, Singapore; ^4^Eye Academic Clinical Program (Eye ACP), Duke NUS Medical School, Singapore, Singapore; ^5^Division of General Surgery (Endocrine & Thyroid Surgery), Department of Surgery, National University Hospital, Singapore, Singapore

**Keywords:** glaucoma, large language models, ChatGPT, artificial intelligence, patient education

## Abstract

**Objective:**

The aim of this study was to evaluate the accuracy, comprehensiveness, and safety of a publicly available large language model (LLM)—ChatGPT in the sub-domain of glaucoma.

**Design:**

Evaluation of diagnostic test or technology.

**Subjects, participants, and/or controls:**

We seek to evaluate the responses of an artificial intelligence chatbot ChatGPT (version GPT-3.5, OpenAI).

**Methods, intervention, or testing:**

We curated 24 clinically relevant questions in the domain of glaucoma. The questions spanned four categories: pertaining to diagnosis, treatment, surgeries, and ocular emergencies. Each question was posed to the LLM and the responses obtained were graded by an expert grader panel of three glaucoma specialists with combined experience of more than 30 years in the field. For responses which performed poorly, the LLM was further prompted to self-correct. The subsequent responses were then re-evaluated by the expert panel.

**Main outcome measures:**

Accuracy, comprehensiveness, and safety of the responses of a public domain LLM.

**Results:**

There were a total of 24 questions and three expert graders with a total number of responses of *n* = 72. The scores were ranked from 1 to 4, where 4 represents the best score with a complete and accurate response. The mean score of the expert panel was 3.29 with a standard deviation of 0.484. Out of the 24 question-response pairs, seven (29.2%) of them had a mean inter-grader score of 3 or less. The mean score of the original seven question-response pairs was 2.96 which rose to 3.58 after an opportunity to self-correct (z-score − 3.27, *p* = 0.001, Mann–Whitney U). The seven out of 24 question-response pairs which performed poorly were given a chance to self-correct. After self-correction, the proportion of responses obtaining a full score increased from 22/72 (30.6%) to 12/21 (57.1%), (*p* = 0.026, χ^2^ test).

**Conclusion:**

LLMs show great promise in the realm of glaucoma with additional capabilities of self-correction. The application of LLMs in glaucoma is still in its infancy, and still requires further research and validation.

## 1 Introduction

In the recent years, there has been great effort into developing large language models (LLMs), large artificial neural networks which leverage on massive datasets to perform a variety of natural language processing tasks. LLMs are increasingly being used in multiple fields, including the field of healthcare (1). For example, some researchers have been exploring how LLMs can be beneficial in providing information to patients in place of traditional FAQs (frequently asked questions) (2).

We seek to evaluate the responses of an artificial intelligence chatbot ChatGPT (version GPT-3.5, OpenAI) (3). ChatGPT is based on a Large Language Model (LLM) and was trained on a massive dataset of text (570 gigabytes worth of data with a model size of 175 billion parameters) (4). Although the response to ChatGPT varied across disciplines, information from ChatGPT is generally viewed as high quality and reliable (5).

However, most LLMs available for public use are based on a general model and are not trained nor fine-tuned specifically for the medical field, let alone a specialty such as ophthalmology. Some recent papers have shown ChatGPT’s potential in passing medical board exam and answer general health queries (6). In ophthalmology, several studies evaluated its performance in ophthalmology board exam, answering queries related to ocular symptoms, and retinal diseases (2, 7, 8). One study compared the performance of ChatGPT in diagnosing glaucoma compared to ophthalmology residents and found the performance comparable between the two groups (9). Overall, these preliminary studies proved promising for ChatGPT as a tool that could be leveraged in the healthcare industry. Nevertheless, to date, none had evaluated its performance in answering queries pertaining to the subspecialty field of glaucoma.

Glaucoma refers to a group of progressive optic neuropathies characterized by optic disc excavation, or cupping, and corresponding patterns of vision loss. It is a common ocular condition with high morbidity. The global prevalence of glaucoma for population aged 40–80 years is 3.54% and the number of people with glaucoma worldwide is predicted to increase to 111.8 million by 2040 (10). Patients with early stages of glaucoma are frequently asymptomatic and patients may have questions regarding screening for the condition, how a diagnosis is made, and questions regarding their treatment options and the associated side effects. With automated intelligence becoming more accessible, patients may use publicly available artificial intelligence chatbots to address their queries around glaucoma (11).

A cross sectional study by Bernstein et al. demonstrated that AI-generated and human responses in the field of ophthalmology can still largely be differentiated with the current generation of LLMs (12). Even outside of ophthalmology, there are studies that show that people prefer chatbot responses over physician responses (5). However, another study reported that the responses provided by ChatGPT required reading comprehension of a higher grade level compared to publicly-available online resources (13).

Hence, the aim of our study was to evaluate how a prototypical LLM, ChatGPT, fares when it comes to queries pertaining to glaucoma, especially with regards to its accuracy, comprehensiveness, and safety of the responses.

## 2 Methods

The publicly available ChatGPT automated intelligence chatbot was accessed at the website.^1^ We used the ChatGPT Mar 23 version for this study. ChatGPT was accessed from 1 March 2023 to 31 March 2023.

Glaucoma specialists (VK, KL, MT) curated a series of 24 clinically relevant questions that are commonly asked by patients. The process of curation included referencing from established resources online namely, the American Academy of Ophthalmology (14) and the National Eye Institute (15). To further draw on questions related to the local context of Singapore, we also curated questions from the “Frequently Asked Questions” section of National University Hospital (16) and Singapore National Eye Centre (17) websites, which are tertiary healthcare institutions from Singapore. The panel of specialists then, based on their extensive experience in their daily interactions with patients in the glaucoma clinic, selected and refined questions which they commonly encountered in a clinical setting. The full list of the 24 curated questions and responses are shown in Supplementary material A1.

These 24 clinically relevant questions spanned 4 categories: pertaining to diagnosis, treatment, surgeries, and ocular emergencies. The questions posed to the chatbot are also known as a “prompt” as per industry parlance. Each prompt was placed into an individual chatbot session as prior conversation could bias subsequent responses by the chatbot. The output of the chatbot for each session was then saved.

The responses were then evaluated by a panel of three glaucoma specialists (LSC, VK, KL) with 19, 7, and 5 years of experience, respectively, in managing patients with glaucoma. Both the query as well as the ChatGPT-generated full response from the chatbot was provided to each specialist separately. The specialists were blinded to each other’s responses. Individually, the specialists were instructed to read the query and the full response from the chatbot before grading each pair using a Likert scale as described in Table 1. In brief, a higher value indicated a more holistic yet accurate response, with 4 denoted as “No inaccuracies, comprehensive response,” and 1 denoted as “Gross inaccuracies with possible threat to patient safety.”

**Table 1:** Rubrics provided to expert graders with corresponding numerical scores.

Score	Description
1	Gross inaccuracies with possible threat to patient safety
2	Major factual inaccuracies without threat to patient safety
3	Minor to no factual inaccuracies, incomplete response (i.e., will benefit from inclusion of other pertinent points)
4	No inaccuracies, comprehensive response.

Given that these are responses to patient-initiated medical questions, there will be potential instances where the chatbot responds in a way that poses a threat to patient safety, for example by giving advice that delays treatment or by advising a patient an incorrect treatment which is harmful or sight-threatening. In such cases (as evaluated by the expert panel), the response would automatically be assigned the lowest score of 1 (as per Table 1).

A mean score of more than 3.0 was deemed to be appropriate while a mean score of 3.0 or less was deemed to be inappropriate.

For responses which were deemed inappropriate, we further prompted the chatbot to self-correct by entering a second prompt*: “This does not seem correct, could you refine your answer?.”* The new responses were collated and presented as question-response pairs to the three expert graders. This second round of grading was performed 4 weeks after the initial grading. The graders were blinded to the exact nature of the prompt in this second round of grading, and were asked to re-evaluate the new answer separately. The full list of questions and responses are provided in Supplementary material A2.

We compared the mean score of the responses before and after self-correction using the Wilcoxon Signed-Rank Test.

Subsequently, we compared holistic (complete and accurate responses) versus those which were lacking in either aspect. More precisely, we compared the rate of complete and accurate responses (score of 4), versus the rate of responses of those with inaccuracies or incompleteness (score of 3 and below). This was compared using a χ2 test.

Statistical analyses were performed using Python (version 3.9.7) along with the following modules: NumPy (version 1.20.3), SciPy (version 1.7.2), pandas (version 1.3.4), and statsmodels (version 0.12.2). *p* < 0.05 was used as a significance threshold.

## 3 Results

There were a total of 24 questions and three expert graders with a total number of responses of *n* = 72. The mean score of the expert panel was 3.29, the mode was 3, and the standard deviation was 0.484. 17 out of 24 (70.8%) questions were graded as appropriate while 7 out of 24 (29.2%) questions were graded as inappropriate by the expert panel. Out of the 7 inappropriate questions, 6 scored 3.0, while 1 response scored 2.67 (Response A7). The basic descriptive statistics are shown in Table 2.

**Table 2:** Basic descriptive statistics from graders.

	Grader A	Grader B	Grader C
n	24	24	24
Median	4	3	3
Mean	3.67	3.17	3.04
Standard deviation	0.48	0.47	0.20

The scores were also grouped based on their categories: diagnosis, treatment, surgeries, and ocular emergencies (Table 3). Questions within the “Diagnosis” group scored the best out of the 4 with a mean score of 3.33 ± 0.55.

**Table 3:** Breakdown of scores by category.

	Diagnosis	Treatment	Surgeries	Ocular emergencies
n	27	27	15	3
Mean	3.33	3.30	3.27	3.00
Standard deviation	0.55	0.47	0.46	0.00 *

*Standard deviation of 0.00 as the response obtained a score of 3 from each of the 3 graders.

Out of the 24 question-response pairs, seven (29.2%) of them had a mean inter-grader score of 3 or less. The mean score of the original seven question-response pairs was 2.96 which rose to 3.58, and a median score which rose from 3.0 to 4.0 (Z = −3.06, *p* = 0.001, Wilcoxon Signed-Rank Test) after ChatGPT was given a chance to self-correct, which achieved statistical significance.

The original question-response pairs obtained a maximal score of four—22 out of 72 times (30.6%). The self-corrected question-response pairs obtained a maximal score 12 out of 21 times (57.1%; *p* = 0.026, χ2 test).

Out of the 72 scores, there was only 1 response which was graded as 2 (Question A7). 23 out of 24 (95.8%) of question-response pairs were rated as 3 or higher by all three experts.

We also performed a qualitative analysis of the seven inappropriate responses in Supplementary material A2, detailing potential areas of weakness and suggestions for improvement. Specifically, the prompt *“My doctor told me I have glaucoma but my eye pressures are normal, how can that be possible?”* was the lowest scoring question as graded by the expert panel. The question and response are both reproduced in Supplementary material A3. The paragraph highlighted in bold was factually incorrect based on the known physiology of glaucoma. The self-corrected paragraph did not include the same factually incorrect information, and in fact, included a section of physiology of normal tension glaucoma, and even included a caveat which qualified that the exact mechanisms behind normal tension glaucoma are not fully understood.

## 4 Discussion

In this study, we demonstrated that large language models show great promise in the realm of glaucoma as 70.8% of question-answer pairs were deemed as appropriate by the expert panel. Automated intelligence chatbots could represent a paradigm shift away from the traditional doctor-patient model. The possibilities of applying LLMs into a healthcare setting are endless, and the authors offer some potential applications towards the end of the discussion section.

This paper is one of the first to investigate LLMs in the context of glaucoma symptomology. Special effort was taken to formulate “real world” questions for the LLM compared to other LLM papers in the literature which have been posing standardised exam questions. This set of curated questions simulated and evaluated more realistic scenarios compared to standardised questions, which potentially allows our findings to be extrapolated to clinical applications.

Large language models also demonstrate a degree of safety in mind as we note that two out of 24 of the model’s responses erred on the side of caution, prefacing its responses with disclaimers such as “As an AI language model, I cannot provide personalised medical advice.” The presence or absence of this disclaimer statement depended on the phrasing of the initial prompt.

Out of the seven inappropriate responses, there was one response in particular with gross factual errors with regards to pathophysiology of disease. These errors arise likely because the LLM was trained on a general model and not dedicated medical datasets. These kinds of errors will be difficult for the lay person to pick up as they require an understanding of the different subtypes of secondary glaucoma. However, this error in pathophysiology does not pose a direct threat to patient safety. Even though the LLM was able to self-correct after prompting, it is unlikely that the general public has the domain knowledge to identify an inaccurate answer for the LLM to revise, thereby posing a potential risk of misinformation. Though, in this specific case, this was a misunderstanding of pathophysiology and is unlikely to cause direct patient harm.

Although such chatbots are unlikely to provide personalised medical advice at this stage, the information that such chatbots provide may be used as a starting point for discussion in the process of informed consent. A study by Gilson et al. showed that ChatGPT performed as well as a third-year medical student on the United States Medical Licensing Examination (USMLE) (6). This is a reminder for clinicians to recognise this new paradigm in the information age and either embrace or work alongside the use of such technology when treating and counselling patients moving ahead.

Additionally, usage of large language models can be used in an automation pipeline to draft responses to a large swath of emails and phone calls that a medical institution might face on the daily. This could potentially reduce many clinician-hours answering important but tedious queries from patients, for example, regarding their medications or post-operative regimen, the responses to which are frequently repetitive in nature. Clinicians can thereafter review the model’s responses, edit the response as appropriate before sending them to patients, thereby significantly speeding up the workflow.

Clinicians should also note the perils of such a technology. Zuccon et al. found evidence that the contents of the user-inputted prompt can deceive the model into providing an incorrect answer to a question that the model could otherwise answer correctly (18)—this could lead to confirmation biases.

We note the potential for ChatGPT to fabricate responses (for example, in the case of Question A7—see Supplementary material A2)—which has the potential of causing harm to patients especially if responses are not vetted adequately by a certified doctor in the field.

Some limitations of this study include the fact that there are no validated rubrics to grade the responses of automated intelligence chatbots. What this paper defined as ground truth was the consensus grading of three glaucoma specialists who are familiar with similar questions in their practice.

Another limitation was that we were unable to evaluate the reliability and sources that ChatGPT used due to the nature of the underlying large language model used. ChatGPT 3.5 was trained on a general large language model and was not specific to medicine. Furthermore, it does not contain demographically specific information.

The small number of graders who responded to this study and the small number of questions is another limitation of this study. This may potentially reduce the generalizability of our results to other patient populations.

The criteria set out in this study prioritised completeness and accuracy of responses. However, it is important to note that there is other criterion that may be of importance as well, such as comprehensibility, when it comes to patient-facing responses.

This study also raised questions for the future. How should clinicians be educated on the potential shortfalls of public usage of chatbots? Are there public health policy implications from a regulator or governmental perspective?

This review article serves as a preview of what the future holds for the use of LLM in the field of glaucoma. Exactly how much further refinement is required before mainstream adoption of this technology by healthcare providers remains to be seen.

## Data availability statement

The original contributions presented in the study are included in the article/Supplementary material, further inquiries can be directed to the corresponding author.

## Author contributions

DT: Conceptualization, Data curation, Formal analysis, Methodology, Writing – original draft, Writing – review & editing. Y-CT: Conceptualization, Investigation, Methodology, Project administration, Supervision, Writing – review & editing. VK: Data curation, Methodology, Supervision, Writing – review & editing. LC: Data curation, Methodology, Supervision, Writing – review & editing. MA: Methodology, Supervision, Writing – review & editing. KL: Data curation, Methodology, Supervision, Writing – review & editing. CC-Y: Methodology, Supervision, Writing – review & editing. KN: Supervision, Writing – review & editing. MT: Conceptualization, Data curation, Formal analysis, Investigation, Methodology, Supervision, Writing – review & editing.

## Footnotes

^1^https://chat.openai.com/
