# Supplementary material for: Evaluating Chatbot responses to patient questions in the field of glaucoma
Source: Front Med (Lausanne). 2024 Jul 9;11:1359073. doi: 10.3389/fmed.2024.1359073 (PMC11267485; doi:10.3389/fmed.2024.1359073)
Supplement: Supplementary file 1 [file Table_1.DOCX]

Supplementary Material

**Supplementary Material A1: Full list of questions and responses from chatbot which were provided to the expert graders**

| **No** | **Question** | **Chat GPT Response** |
| --- | --- | --- |
| **Diagnosis** | | |
| A1 | What are the symptoms of glaucoma? | Glaucoma is a group of eye diseases that can cause damage to the optic nerve and vision loss. The symptoms of glaucoma can vary depending on the type and severity of the disease, but in the early stages, there may be no noticeable symptoms. However, as the disease progresses, some common symptoms may include:  1. Loss of peripheral vision: This is often the first symptom of glaucoma. You may notice a gradual loss of your side vision, which can progress to tunnel vision over time.  2. Blurred vision: You may notice that your vision is becoming increasingly blurry or hazy.  3. Halos around lights: You may see halos or rainbow-colored rings around lights, especially at night.  4. Eye pain or discomfort: You may experience pain or discomfort in one or both eyes, as well as headaches.  5. Nausea and vomiting: In some cases, glaucoma can cause nausea and vomiting, particularly when the pressure inside the eye is very high.  If you experience any of these symptoms, it is important to see an eye doctor for an evaluation. Early detection and treatment of glaucoma can help prevent vision loss and preserve your eyesight. |
| A2 | How is glaucoma diagnosed? | Glaucoma is a group of eye diseases that cause damage to the optic nerve and can lead to vision loss or blindness. There are different types of glaucoma, and the diagnostic process can vary depending on the type and severity of the condition. However, in general, the diagnosis of glaucoma involves the following:  1. Eye examination: A comprehensive eye examination is typically the first step in diagnosing glaucoma. This may include tests such as tonometry, which measures the pressure inside the eye, and visual field testing, which checks for any areas of vision loss.  2. Optic nerve assessment: The appearance of the optic nerve is examined by an ophthalmologist or optometrist. They will look for any signs of damage or changes to the optic nerve, such as thinning or cupping.  3. Imaging tests: Additional tests may be performed to provide more detailed images of the optic nerve and the structures inside the eye. These may include optical coherence tomography (OCT) or scanning laser polarimetry (SLP).  4. Medical history: The doctor may also ask about your medical history and any family history of eye problems or glaucoma.  If glaucoma is diagnosed, treatment may involve medication, laser therapy, or surgery, depending on the type and severity of the condition. Regular follow-up appointments will also be necessary to monitor the progression of the disease and adjust the treatment plan as needed. |
| A3 | My doctor diagnosed me with glaucoma, what does that mean for me, and will I go blind? | Glaucoma is a condition that affects the optic nerve, which is responsible for transmitting visual information from the eye to the brain. It is typically caused by a buildup of pressure within the eye, which can damage the optic nerve over time.  If left untreated, glaucoma can lead to vision loss and, in severe cases, blindness. However, with early diagnosis and treatment, the progression of the disease can often be slowed or stopped, and many people with glaucoma are able to maintain their vision.  Treatment for glaucoma may involve prescription eye drops, oral medications, or in some cases, surgery. Your doctor will work with you to develop a treatment plan that is tailored to your specific needs.  It is important to follow your doctor's recommendations for monitoring and treating your glaucoma to help prevent further damage to your vision. Regular eye exams are also important to monitor the progression of the disease and adjust treatment as needed. |
| A4 | My doctor diagnosed me with glaucoma but I feel that my vision is not impaired, is the diagnosis wrong? | Glaucoma is a condition that damages the optic nerve, which can lead to vision loss if left untreated. However, in the early stages of the disease, it is possible to have glaucoma and not experience any noticeable symptoms or changes in vision. Therefore, it is possible that your diagnosis is correct, even if you feel that your vision is not currently impaired.  It is important to follow up with your doctor and discuss any concerns or questions you may have about your diagnosis. Your doctor may perform additional tests or exams to confirm the diagnosis and determine the best course of treatment for your individual needs. It is also important to follow any recommended treatment plan, as early intervention and treatment can help to slow or prevent further vision loss. |
| A5 | My parents and siblings have glaucoma, am I at risk for glaucoma and should I go for screening for glaucoma too? | Yes, having a family history of glaucoma puts you at an increased risk of developing the condition. It is recommended that you undergo regular eye examinations to screen for glaucoma, particularly if you are over the age of 40 or have other risk factors such as high eye pressure, diabetes, or high myopia.  During a glaucoma screening, your eye doctor will perform several tests to check your eye pressure, examine your optic nerve, and assess your visual field. If any abnormalities are detected, further testing and evaluation may be necessary.  It's important to remember that early detection and treatment are crucial in preventing vision loss from glaucoma. Therefore, if you have a family history of glaucoma, I would recommend that you schedule a comprehensive eye exam with an eye doctor as soon as possible to assess your risk and determine if any further testing or treatment is needed. |
| A6 | My doctor told me that I have raised eye pressures, does that mean that I have glaucoma? | Raised eye pressure, also known as intraocular pressure (IOP), can be a risk factor for developing glaucoma, but it does not necessarily mean that you have glaucoma. Glaucoma is a condition where the optic nerve that connects the eye to the brain is damaged, often due to increased eye pressure, which can lead to vision loss if left untreated. However, not everyone with elevated eye pressure will develop glaucoma.  Your doctor may recommend further tests, such as a visual field test and optic nerve evaluation, to determine whether you have glaucoma or if you are at risk of developing it. It's essential to follow your doctor's recommendations for regular eye exams to monitor your eye pressure and detect any changes in your vision. If you do have glaucoma, early detection and treatment can help prevent or slow down the progression of vision loss. |
| A7 | My doctor told me I have glaucoma but my eye pressures are normal, how can that be possible? | It is possible to have glaucoma even if your intraocular pressures (IOP) are within the normal range. In fact, a certain type of glaucoma, called normal-tension glaucoma, is characterized by the presence of glaucomatous damage to the optic nerve and visual field loss despite having normal IOP levels.  There are also other types of glaucoma that may not be related to IOP at all, such as congenital glaucoma or pigmentary glaucoma. These conditions may be caused by other factors, such as structural abnormalities or blockages in the eye's drainage system.  Your doctor may perform additional tests, such as a visual field test or optic nerve evaluation, to confirm a diagnosis of glaucoma and determine the best course of treatment. It is important to follow up with your doctor and discuss any concerns or questions you may have about your condition. |
| A8 | I do not have a family history of glaucoma. What is the likelihood of me getting glaucoma? | The risk of developing glaucoma can depend on several factors, including age, ethnicity, and certain medical conditions. While having a family history of glaucoma is a known risk factor, it is not the only factor that can increase your chances of developing the condition.  In general, the likelihood of developing glaucoma increases as you get older. Additionally, certain ethnic groups, such as African Americans, Hispanics, and Asians, have a higher risk of developing certain types of glaucoma.  It is also important to note that glaucoma can develop without any symptoms and go undetected until it causes significant vision loss. Therefore, regular eye exams are important, particularly as you age, to detect and treat any eye conditions early on.  If you have concerns about your risk of developing glaucoma, it is always best to consult with an eye doctor who can assess your individual risk factors and provide personalized recommendations. |
| A9 | I was diagnosed with glaucoma 4 years ago but missed my appointments due to COVID, how do I know if my condition is worsening? | Glaucoma is a serious eye condition that can lead to vision loss or blindness if left untreated. If you have missed your appointments due to COVID, it is important to schedule an eye exam with your eye doctor as soon as possible to assess the status of your glaucoma.  During your eye exam, your eye doctor will perform various tests to check your eye pressure, assess the optic nerve, and check for any vision changes. If your eye pressure is higher than normal or if there are any changes in your optic nerve or visual field, your eye doctor may recommend changes to your treatment plan to prevent further damage to your vision.  In the meantime, if you experience any changes in your vision, such as blurred vision, loss of peripheral vision, or difficulty seeing in low light conditions, it is important to contact your eye doctor right away. These symptoms could be a sign that your glaucoma is worsening and immediate medical attention is necessary to prevent further vision loss. |
| **Treatment** | | |
| B1 | What are the treatment options for glaucoma? | Glaucoma is a group of eye diseases that can lead to damage of the optic nerve and vision loss if left untreated. The treatment options for glaucoma depend on the type and severity of the condition, and may include:  1. Eye drops: Glaucoma eye drops are often the first line of treatment for the condition. They work by reducing the production of fluid in the eye or increasing the drainage of fluid. Examples of glaucoma eye drops include prostaglandin analogs, beta blockers, alpha agonists, and carbonic anhydrase inhibitors.  2. Oral medications: In some cases, oral medications may be prescribed to help reduce intraocular pressure.  3. Laser therapy: Laser therapy, such as selective laser trabeculoplasty (SLT) or laser peripheral iridotomy (LPI), can help reduce intraocular pressure by improving the drainage of fluid from the eye.  4. Surgery: Surgical options for glaucoma include trabeculectomy, which involves creating a small opening in the eye to allow fluid to drain out, and shunt surgery, which involves implanting a small tube to help drain fluid.  It's important to note that while treatment can slow or halt the progression of glaucoma, it cannot reverse vision loss that has already occurred. Early detection and treatment are critical in managing the condition and preventing further vision loss. Regular eye exams are recommended to detect glaucoma in its early stages. |
| B2 | What do I do if I have eye redness after starting a glaucoma eyedrop? | If you experience eye redness after starting a glaucoma drop, it is important to contact your eye doctor or healthcare provider as soon as possible. Eye redness can be a sign of an allergic reaction or an adverse reaction to the medication.  In some cases, the eye redness may be a temporary side effect that will go away on its own after a few days. However, it is important to seek medical attention to ensure that your condition is properly evaluated and managed.  Your doctor may recommend discontinuing the use of the glaucoma drop or switching to a different medication that is better tolerated by your eyes. In some cases, additional treatments or medications may be necessary to manage the symptoms of an allergic or adverse reaction.  It is important to never stop taking a medication without first consulting with your healthcare provider. They will be able to provide you with the best course of action for your individual situation. |
| B3 | I was diagnosed with glaucoma and given eye drops to use but I don't feel any improvement, can I stop my glaucoma eye drops? | As an AI language model, I cannot provide medical advice, diagnosis, or treatment. Therefore, you should always consult with your ophthalmologist or healthcare provider before making any changes to your medication regimen.  It's important to note that glaucoma is a chronic condition that requires ongoing management, and treatment typically involves a combination of medications, surgical procedures, or both. It can take several weeks or even months to see improvements in your vision or intraocular pressure after starting glaucoma eye drops.  Stopping or changing medications without the advice of your healthcare provider can have serious consequences, including worsening of your glaucoma and potential vision loss. If you're experiencing side effects or have concerns about the effectiveness of your current treatment, talk to your ophthalmologist or healthcare provider about adjusting your treatment plan. |
| B4 | My doctor started me on latanoprost for glaucoma, what are the side effects that I should be looking out for? | Latanoprost is a medication used to treat glaucoma and high eye pressure. Some of the side effects that you should be aware of when taking latanoprost include:  1. Redness and itching of the eye  2. Changes in the color of the iris (the colored part of the eye)  3. Increased growth and thickness of the eyelashes  4. Sensitivity to light  5. Blurred vision or other changes in vision  6. Eye pain  7. Burning or stinging sensation in the eye  8. Dryness or watering of the eye  9. Discharge or crusting around the eye  10. Headache  11. Nausea  If you experience any of these side effects or any other unusual symptoms, you should contact your doctor right away. It is important to keep all follow-up appointments with your doctor while taking latanoprost to monitor your eye pressure and ensure that the medication is working properly. |
| B5 | My doctor started me on glaucoma eyedrops, what should I do if I miss one dose? | It is important to take glaucoma eye drops exactly as prescribed by your doctor in order to effectively manage the condition and prevent further damage to your eyesight. However, if you do happen to miss a dose, here are some general guidelines:  1. If you remember the missed dose within a few hours of the scheduled time, go ahead and apply the drop as usual.  2. If you remember the missed dose closer to the time of the next scheduled dose, skip the missed dose and resume your normal dosing schedule.  3. Do not double up on doses to make up for a missed one, as this can increase the risk of side effects.  If you are unsure about what to do or have any concerns, it is always best to consult with your doctor or pharmacist for guidance. They can provide you with specific instructions based on your individual treatment plan and health history. |
| B6 | My doctor started me on glaucoma eyedrops, my next appointment is in one month but I have run out of medicine, can I wait until the next appointment to get more eyedrops? | As an AI language model, I cannot provide medical advice. However, it is important to note that glaucoma is a serious eye condition that can cause permanent vision loss if left untreated. If you have run out of your glaucoma eye drops, it is important to contact your doctor's office or the pharmacy as soon as possible to request a refill or ask for guidance on what to do next. Your doctor may be able to provide you with a prescription refill over the phone, or they may recommend that you come in for an earlier appointment to get a new prescription. It is important to follow your doctor's instructions and not wait until your next appointment if you have run out of your medication. |
| B7 | I was told that I require laser peripheral iridotomy for narrow angles, what if I choose not to go for the procedure? | Laser peripheral iridotomy is a procedure used to treat narrow angles, a condition in which the drainage system for fluid within the eye becomes blocked. If left untreated, this can lead to an increase in eye pressure and potentially cause permanent damage to the optic nerve and vision loss.  It's important to understand the potential risks of not undergoing the recommended treatment. Without treatment, you may experience increased eye pressure and a risk of developing glaucoma, a condition that damages the optic nerve and can cause vision loss. Additionally, untreated narrow angles can cause eye pain, headaches, and other symptoms.  If you have concerns about the procedure, it's important to discuss them with your doctor and ask any questions you may have. They can provide you with information on the benefits and risks of the procedure, as well as alternative treatment options if available.  Ultimately, the decision to undergo laser peripheral iridotomy or not is up to you. However, it's important to consider the potential consequences of not treating your narrow angles and to discuss your concerns with your doctor before making a decision. |
| B8 | I was recently diagnosed with glaucoma and the doctor offered selective laser trabeculoplasty versus topical pressure lowering eyedrops, what are the pros and cons of each option? | Glaucoma is a condition that causes damage to the optic nerve and can result in vision loss. One of the primary goals of treatment is to lower the intraocular pressure (IOP) in order to slow down or prevent further damage to the optic nerve. Selective laser trabeculoplasty (SLT) and topical pressure lowering eyedrops are two commonly used treatments for glaucoma. Here are some pros and cons of each option:  Selective laser trabeculoplasty (SLT):  Pros:  - Non-invasive procedure  - No need for daily eye drops  - Can lower IOP by about 20-30% on average  - Can be repeated if needed  - Minimal risk of side effects  Cons:  - May not work for everyone  - Can take several weeks to see results  - May need additional treatment with eye drops or other procedures  Topical pressure lowering eyedrops:  Pros:  - Effective at lowering IOP in most cases  - Can be customized to individual patient needs  - Can be used in combination with other medications  - Can work quickly (within hours)  Cons:  - Must be used daily for long-term management  - Risk of side effects such as redness, itching, and stinging  - Some eye drops can cause systemic side effects (e.g. beta-blockers may affect heart rate and blood pressure)  - Cost may be a factor, especially if multiple eye drops are needed  Ultimately, the choice between SLT and topical pressure lowering eyedrops depends on various factors such as the severity of the glaucoma, patient preference, and potential side effects. Your doctor can help guide you in making the best decision for your specific case. |
| B9 | I have a history of glaucoma on latanoprost, I have read about a new class of medicine called the rho-kinase inhibitors and would like to switch to the new medicine, what are the side effects? | As an AI language model, I cannot provide personalized medical advice. However, I can give you general information about rho-kinase inhibitors and their potential side effects.  Rho-kinase inhibitors are a new class of medication used in the treatment of glaucoma. They work by relaxing the muscles in the eye's drainage system, allowing for better fluid outflow and reducing intraocular pressure.  Like any medication, rho-kinase inhibitors can have potential side effects, although they may differ from person to person. Some common side effects associated with rho-kinase inhibitors include:  - Eye irritation or redness  - Blurred vision  - Headaches  - Cold-like symptoms, such as a runny nose or sore throat  Less common side effects may include:  - Dry eyes  - Changes in taste or smell  - Eye pain  - Eye discharge or crusting  - Light sensitivity  It is essential to talk to your doctor before making any changes to your medication regimen. Your doctor will be able to evaluate your individual situation and determine whether switching to a rho-kinase inhibitor is appropriate for you. They can also discuss any potential side effects and how to manage them if they occur. |
| **Surgery** | | |
| C1 | Why does my vision not improve after glaucoma surgery? | There can be several reasons why your vision may not improve after glaucoma surgery:  1. Damage already done: If the glaucoma has caused significant damage to your optic nerve before the surgery, it may not be possible to restore your vision to its previous level. The surgery can only help to prevent further damage.  2. Complications: Sometimes, complications may occur during or after the surgery, which can lead to vision problems. These can include bleeding, infection, or inflammation.  3. Cataracts: Glaucoma surgery can sometimes cause or accelerate the development of cataracts, which can also affect your vision.  4. Time: It can take several weeks or even months for your vision to fully stabilize after glaucoma surgery. Your doctor may need to adjust your medications or recommend additional treatments during this time.  5. Underlying conditions: Other underlying conditions, such as diabetes or high blood pressure, can also affect your vision and may need to be managed in conjunction with your glaucoma treatment.  It's important to discuss any concerns about your vision with your ophthalmologist, who can provide a more specific explanation based on your individual circumstances. |
| C2 | I was diagnosed with angle closure and the glaucoma doctor asked me to consider cataract surgery even though I can see well, should I listen to his advice? | As an AI language model, I cannot provide medical advice. However, I can offer some general information about angle-closure and cataract surgery.  Angle-closure occurs when the drainage angle between the iris and cornea is closed or obstructed, leading to a buildup of pressure in the eye. This condition can cause damage to the optic nerve, which can eventually lead to vision loss. Cataracts, on the other hand, occur when the natural lens of the eye becomes cloudy, leading to vision problems.  Cataract surgery is often recommended for people with both angle-closure and cataracts because the procedure can help to improve the drainage of fluid in the eye, which can lower eye pressure and reduce the risk of optic nerve damage. Cataract surgery can also remove the cloudy lens and replace it with a clear artificial lens, which can improve vision.  It's important to talk to your doctor about the risks and benefits of cataract surgery in your specific case, especially if you are currently experiencing good vision. Your doctor can help you determine whether cataract surgery is the best course of action for your individual needs and circumstances. |
| C3 | My glaucoma doctor offered either a tube surgery or trabeculectomy surgery for me, what are the differences? | Both tube surgery and trabeculectomy are surgical procedures that can be used to treat glaucoma when medications and other less invasive interventions are no longer effective in controlling the disease.  Trabeculectomy is the more established of the two procedures and has been performed for several decades. During trabeculectomy, the surgeon creates a small opening in the sclera (the white part of the eye) and removes a small piece of tissue from the eye's drainage system to create a new drainage pathway for the aqueous humor, the fluid inside the eye. This allows the fluid to flow out of the eye, reducing intraocular pressure (IOP).  In tube surgery, the surgeon implants a small tube, called a shunt or a stent, in the eye. The tube is inserted through a tiny incision in the eye and is placed to drain excess fluid from the eye. The tube is connected to a small reservoir or plate that is placed on the surface of the eye under the conjunctiva (the clear covering over the sclera).  The main difference between the two procedures is the way in which they create a new pathway for the aqueous humor to drain out of the eye. Trabeculectomy involves removing tissue from the eye, while tube surgery involves implanting a tube or shunt.  Both procedures can be effective in lowering IOP, but they also carry risks and potential complications. Your doctor will evaluate your specific condition and discuss the benefits and risks of each procedure to determine which is the best option for you. |
| C4 | I am going for a routine cataract surgery but my doctor told me that I should do MIGS surgery at the same time, is that recommended? | MIGS (Minimally Invasive Glaucoma Surgery) is a type of surgical procedure that is performed to treat glaucoma, a condition that causes damage to the optic nerve and can lead to blindness if left untreated. It is usually recommended for patients who have both cataracts and glaucoma.  If your doctor has recommended that you undergo MIGS surgery at the same time as your cataract surgery, it is likely because they believe that you would benefit from the procedure. MIGS surgery is a safe and effective way to treat glaucoma while also addressing cataracts.  However, it is important to understand that every patient is unique, and your individual circumstances will need to be considered by your doctor. You should discuss any concerns you may have about the surgery with your doctor, and ask them to explain the benefits and risks associated with MIGS surgery in your specific case. |
| C5 | I have eye pain and discharge after glaucoma surgery, what should I do? | If you are experiencing eye pain and discharge after glaucoma surgery, it is important to seek medical attention as soon as possible. These symptoms may indicate an infection or other complication that needs prompt treatment.  You should contact your ophthalmologist or the surgeon who performed your glaucoma surgery immediately to report your symptoms and schedule an appointment for an evaluation. They may want to examine your eye and take a sample of the discharge to determine the cause of your symptoms and recommend appropriate treatment.  In the meantime, avoid rubbing or touching your eye, and follow any instructions provided by your doctor. It is also important to keep the area around your eye clean and dry to prevent further irritation or infection.  If you experience any additional symptoms, such as vision changes or severe pain, seek emergency medical attention right away. |
| **Ocular emergencies** | | |
| D1 | A doctor told me that I was a glaucoma suspect previously, what does it mean when I have sudden eye redness, pain, and blurring of vision and should I see a doctor immediately? | If you were told that you were a glaucoma suspect previously and you are now experiencing sudden eye redness, pain, and blurring of vision, it is important to see a doctor immediately. These symptoms may indicate acute angle-closure glaucoma, which is a medical emergency and requires immediate treatment.  Acute angle-closure glaucoma occurs when the pressure inside the eye increases rapidly due to a sudden blockage of fluid drainage. This can cause severe eye pain, redness, and blurred vision. If left untreated, it can lead to irreversible vision loss.  It is essential to seek immediate medical attention if you experience these symptoms to prevent permanent damage to your vision. Your doctor will perform a comprehensive eye exam and may recommend further tests to diagnose and treat your condition.  In addition to seeking medical attention, it is also important to inform your doctor about your previous history of being a glaucoma suspect. This information will help your doctor better understand your overall eye health and make appropriate recommendations for treatment and follow-up care. |
