# Supplementary material for: Evaluating Chatbot responses to patient questions in the field of glaucoma
Source: Front Med (Lausanne). 2024 Jul 9;11:1359073. doi: 10.3389/fmed.2024.1359073 (PMC11267485; doi:10.3389/fmed.2024.1359073)
Supplement: Supplementary file 2 [file Table_2.DOCX]

Supplementary Material

**Supplementary Material A2: Inappropriate responses with highlights and comments**

| **No** | **Question** | **Chat GPT Response** | **Comments** |
| --- | --- | --- | --- |
| A7 | My doctor told me I have glaucoma but my eye pressures are normal, how can that be possible? | It is possible to have glaucoma even if your intraocular pressures (IOP) are within the normal range. In fact, a certain type of glaucoma, called normal-tension glaucoma, is characterized by the presence of glaucomatous damage to the optic nerve and visual field loss despite having normal IOP levels.  ***There are also other types of glaucoma that may not be related to IOP at all, such as congenital glaucoma or pigmentary glaucoma. These conditions may be caused by other factors, such as structural abnormalities or blockages in the eye's drainage system.***  Your doctor may perform additional tests, such as a visual field test or optic nerve evaluation, to confirm a diagnosis of glaucoma and determine the best course of treatment. It is important to follow up with your doctor and discuss any concerns or questions you may have about your condition. | Factually incorrect based on the known physiology of glaucoma.  Intraocular pressure (IOP) is likely to be high in both congenital and pigmentary glaucoma. |
| B3 | I was diagnosed with glaucoma and given eye drops to use but I don't feel any improvement, can I stop my glaucoma eye drops? | As an AI language model, I cannot provide medical advice, diagnosis, or treatment. Therefore, you should always consult with your ophthalmologist or healthcare provider before making any changes to your medication regimen.  It's important to note that glaucoma is a chronic condition that requires ongoing management, and treatment typically involves a combination of medications, surgical procedures, or both. ***It can take several weeks or even months to see improvements in your vision*** or intraocular pressure after starting glaucoma eye drops.  Stopping or changing medications without the advice of your healthcare provider can have serious consequences, including worsening of your glaucoma and potential vision loss. If you're experiencing side effects or have concerns about the effectiveness of your current treatment, talk to your ophthalmologist or healthcare provider about adjusting your treatment plan. | Factually incorrect as glaucoma eye drops does not function to improve vision. Established glaucomatous optic nerve damage does not reverse with correction of intraocular pressures. |
| B7 | I was told that I require laser peripheral iridotomy for narrow angles, what if I choose not to go for the procedure? | Laser peripheral iridotomy is a procedure used to treat narrow angles, a condition in which the drainage system for fluid within the eye becomes blocked. If left untreated, this can lead to an increase in eye pressure and potentially cause permanent damage to the optic nerve and vision loss.  It's important to understand the potential risks of not undergoing the recommended treatment. Without treatment, you may experience increased eye pressure and a risk of developing glaucoma, a condition that damages the optic nerve and can cause vision loss. Additionally, untreated narrow angles can cause eye pain, headaches, and other symptoms.  If you have concerns about the procedure, it's important to discuss them with your doctor and ask any questions you may have. They can provide you with information on the benefits and risks of the procedure, as well as alternative treatment options if available.  Ultimately, the decision to undergo laser peripheral iridotomy or not is up to you. However, it's important to consider the potential consequences of not treating your narrow angles and to discuss your concerns with your doctor before making a decision. | No particularly incorrect statement, however, this response is overly padded with defensive language which does not provide value-add to the user.  Instead, the response could be improved by providing more information on what the laser peripheral iridotomy entails, the risks, benefits, and the alternatives.  Some alternatives might include close monitoring with follow-up visits every 6-9 months with acute angle closure advice, versus clear lens extraction (EAGLE study), or cataract extraction. |
| B8 | I was recently diagnosed with glaucoma and the doctor offered selective laser trabeculoplasty versus topical pressure lowering eyedrops, what are the pros and cons of each option? | Glaucoma is a condition that causes damage to the optic nerve and can result in vision loss. One of the primary goals of treatment is to lower the intraocular pressure (IOP) in order to slow down or prevent further damage to the optic nerve. Selective laser trabeculoplasty (SLT) and topical pressure lowering eyedrops are two commonly used treatments for glaucoma. Here are some pros and cons of each option:  Selective laser trabeculoplasty (SLT):  Pros:  - Non-invasive procedure  - No need for daily eye drops  - Can lower IOP by about 20-30% on average  - Can be repeated if needed  - Minimal risk of side effects  Cons:  - May not work for everyone  - Can take several weeks to see results  - May need additional treatment with eye drops or other procedures  Topical pressure lowering eyedrops:  Pros:  - Effective at lowering IOP in most cases  - Can be customized to individual patient needs  - Can be used in combination with other medications  - Can work quickly (within hours)  Cons:  - Must be used daily for long-term management  - Risk of side effects such as redness, itching, and stinging  - Some eye drops can cause systemic side effects (e.g. beta-blockers may affect heart rate and blood pressure)  - Cost may be a factor, especially if multiple eye drops are needed  Ultimately, the choice between SLT and topical pressure lowering eyedrops depends on various factors such as the severity of the glaucoma, patient preference, and potential side effects. Your doctor can help guide you in making the best decision for your specific case. | This response is fairly comprehensive, however, the section on pros for SLT mentioned a minimal risk of side effects without quantifying the frequency or providing examples, which downplays the potential side effects of SLT.  Secondly, although it is true that the cumulative cost of eyedrops is likely higher over the course of the disease, this is balanced against the high initial outlay for a laser procedure which some patients may face financial difficulties with (which was not highlighted in this response). |
| C2 | I was diagnosed with angle closure and the glaucoma doctor asked me to consider cataract surgery even though I can see well, should I listen to his advice? | As an AI language model, I cannot provide medical advice. However, I can offer some general information about angle-closure and cataract surgery.  Angle-closure occurs when the drainage angle between the iris and cornea is closed or obstructed, leading to a buildup of pressure in the eye. This condition can cause damage to the optic nerve, which can eventually lead to vision loss. Cataracts, on the other hand, occur when the natural lens of the eye becomes cloudy, leading to vision problems.  Cataract surgery is often recommended for people with both angle-closure and cataracts because the procedure can help to improve the drainage of fluid in the eye, which can lower eye pressure and reduce the risk of optic nerve damage. Cataract surgery can also remove the cloudy lens and replace it with a clear artificial lens, which can improve vision.  It's important to talk to your doctor about the risks and benefits of cataract surgery in your specific case, especially if you are currently experiencing good vision. Your doctor can help you determine whether cataract surgery is the best course of action for your individual needs and circumstances. | This response is factually accurate; however, it may be improved by going into detail as to how cataract surgery may improve the drainage of fluid in the eye (which is likely not immediately obvious to non-ophthalmologists). |
| C4 | I am going for a routine cataract surgery but my doctor told me that I should do MIGS surgery at the same time, is that recommended? | MIGS (Minimally Invasive Glaucoma Surgery) is a type of surgical procedure that is performed to treat glaucoma, a condition that causes damage to the optic nerve and can lead to blindness if left untreated. It is usually recommended for patients who have both cataracts and glaucoma.  If your doctor has recommended that you undergo MIGS surgery at the same time as your cataract surgery, it is likely because they believe that you would benefit from the procedure. MIGS surgery is a safe and effective way to treat glaucoma while also addressing cataracts.  However, it is important to understand that every patient is unique, and your individual circumstances will need to be considered by your doctor. You should discuss any concerns you may have about the surgery with your doctor, and ask them to explain the benefits and risks associated with MIGS surgery in your specific case. | Some factual inaccuracy – the response conflates two categories of surgeries: minimally invasive glaucoma surgery (MIGS) and cataract surgery.  MIGS itself does not address cataracts. |
| D1 | A doctor told me that I was a glaucoma suspect previously, what does it mean when I have sudden eye redness, pain, and blurring of vision and should I see a doctor immediately? | If you were told that you were a glaucoma suspect previously and you are now experiencing sudden eye redness, pain, and blurring of vision, it is important to see a doctor immediately. These symptoms may indicate acute angle-closure glaucoma, which is a medical emergency and requires immediate treatment.  Acute angle-closure glaucoma occurs when the pressure inside the eye increases rapidly due to a sudden blockage of fluid drainage. This can cause severe eye pain, redness, and blurred vision. If left untreated, it can lead to irreversible vision loss.  It is essential to seek immediate medical attention if you experience these symptoms to prevent permanent damage to your vision. Your doctor will perform a comprehensive eye exam and may recommend further tests to diagnose and treat your condition.  In addition to seeking medical attention, it is also important to inform your doctor about your previous history of being a glaucoma suspect. This information will help your doctor better understand your overall eye health and make appropriate recommendations for treatment and follow-up care. | There are no factual errors in this response. Possible ways to improve the comprehensiveness of the response would be to include other associated symptoms with acute angle closure: nausea, vomiting, and frontal headache. |
