# Supplementary material for: Evaluating Chatbot responses to patient questions in the field of glaucoma
Source: Front Med (Lausanne). 2024 Jul 9;11:1359073. doi: 10.3389/fmed.2024.1359073 (PMC11267485; doi:10.3389/fmed.2024.1359073)
Supplement: Supplementary file 3 [file Table_3.DOCX]

Supplementary Material

**Supplementary Material A3: Inappropriate response by the large language model**

|  | *“My doctor told me I have glaucoma but my eye pressures are normal, how can that be possible?”.* |
| --- | --- |
| **Inappropriate** | **Initial response**  It is possible to have glaucoma even if your intraocular pressures (IOP) are within the normal range. In fact, a certain type of glaucoma, called normal-tension glaucoma, is characterized by the presence of glaucomatous damage to the optic nerve and visual field loss despite having normal IOP levels.  ***There are also other types of glaucoma that may not be related to IOP at all, such as congenital glaucoma or pigmentary glaucoma. These conditions may be caused by other factors, such as structural abnormalities or blockages in the eye's drainage system.***  Your doctor may perform additional tests, such as a visual field test or optic nerve evaluation, to confirm a diagnosis of glaucoma and determine the best course of treatment. It is important to follow up with your doctor and discuss any concerns or questions you may have about your condition. |
| **Appropriate** | **After allowing the chatbot to self-correct**  Glaucoma is a group of eye conditions that can cause damage to the optic nerve, resulting in vision loss. The primary risk factor for glaucoma is elevated intraocular pressure (IOP), which is typically associated with the disease. However, it is important to note that not all cases of glaucoma are solely dependent on high IOP. There are different types of glaucoma, and some can occur even when IOP remains within the normal range.  Normal-tension glaucoma (NTG) is a subtype of glaucoma in which optic nerve damage and vision loss occur despite normal IOP readings. The exact mechanisms behind NTG are not fully understood, but it is believed that other factors, such as blood flow abnormalities or increased sensitivity of the optic nerve to IOP, may contribute to the development of glaucoma.  In such cases, diagnosing glaucoma becomes more challenging because IOP measurements alone may not be sufficient to identify the condition. Additional diagnostic tests, such as visual field testing, optic nerve imaging, and assessing the optic nerve's appearance, are crucial for a comprehensive evaluation.  If your doctor has diagnosed you with glaucoma despite normal IOP readings, it is likely that they have considered these other factors and performed further examinations to confirm the diagnosis. It is important to follow up with your doctor regularly, adhere to the recommended treatment plan, and ask any additional questions you may have about your condition. |
